# Gene model for the ortholog of *Gar1* in *Drosophila willistoni*

**DOI:** 10.17912/micropub.biology.001818

**Published:** 2025-10-27

**Authors:** Nora Doughty, Ethan Pini, Alizabeth Tobin, Mia Lacenere, Allison Rea, Taylor Holt, Christopher Park, Amy Jones, Michael S. Foulk, Christopher D. Shaffer

**Affiliations:** 1 Washington University in St. Louis, St. Louis, Missouri, United States; 2 Mercyhurst University, Erie, Pennsylvania, United States; 3 The University of Texas at San Antonio, San Antonio, Texas, United States

## Abstract

The *Drosophila willistoni* feature with NCBI Gene ID 6651170 was established to be orthologous to *Drosophila melanogaster* gene *Gar1* . The *Gar1* protein contains the conserved domain H/ACA_rnp_Gar1/Naf1 (InterPro accession IPR007504) and the N-terminus and C-terminus of the protein are glycine-rich. There are major changes in the gene structure of the ortholog, Gar1-PA, between *D. melanogaster* and *D. willistoni* , which have three and four coding exons respectively. Notably, none of the intron positions are conserved between the two species.

**Figure 1. Supporting evidence for the annotation of the Gar1 ortholog in D. willistoni:**
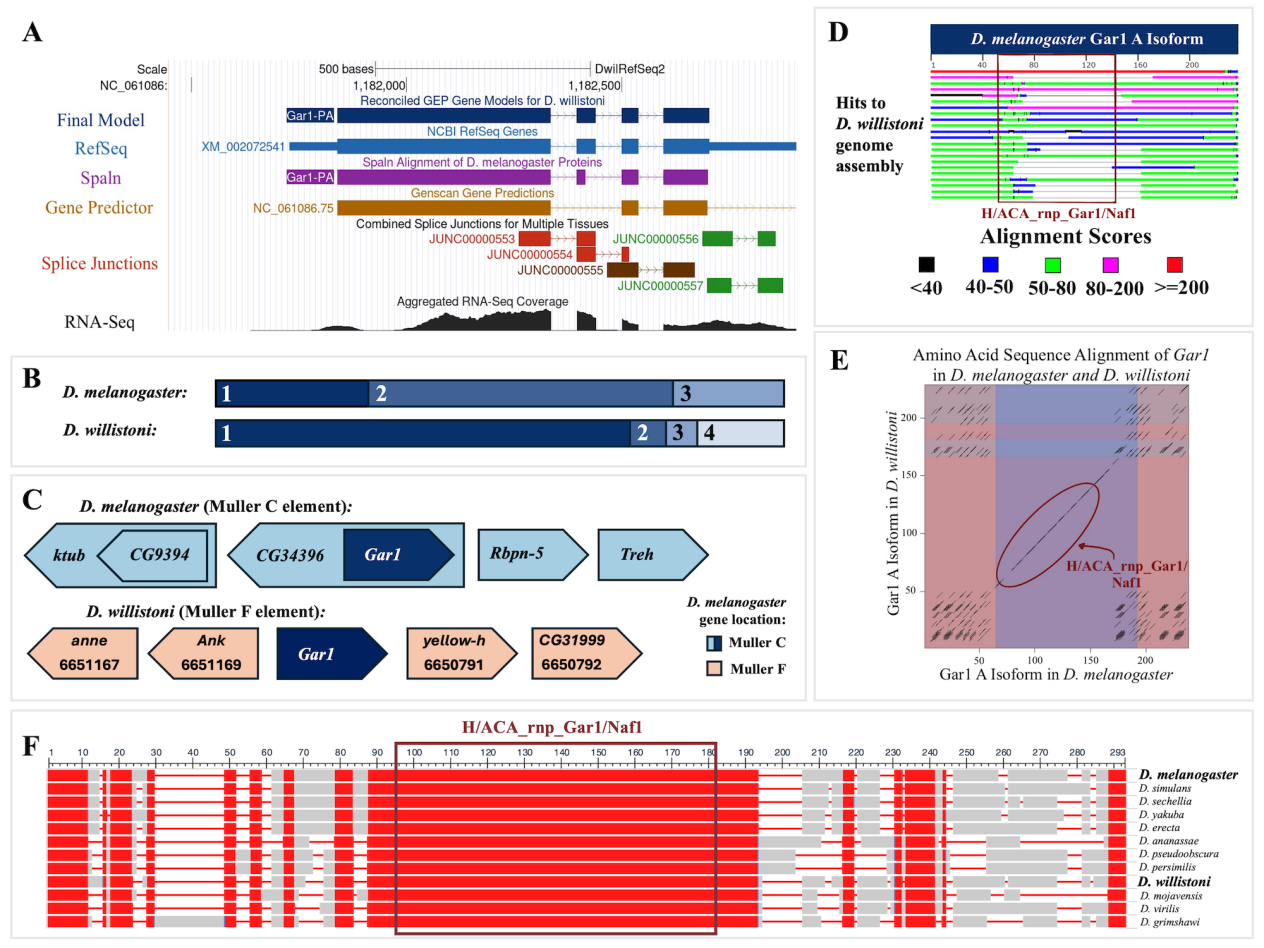
**
(A) *Gar1* gene model in UCSC Genome Browser UCl_dwil_1.1/DwilRefSeq2 assembly with evidence tracks.** The dark blue gene model labeled “Final Model” represents the final reconciled gene model for Gar1-PA, with four coding exons. The other evidence tracks were used to generate the *Gar1* model. From top to bottom: NCBI Reference Sequence (RefSeq) mRNA prediction XM_002072541 (light blue), Spaln alignment against *D. melanogaster* Gar1-PA (purple), Genscan computational gene prediction (brown), combined splice junctions (filtered so that only junctions with scores 10 or above are shown), and aggregated RNA-Seq coverage for *D. willistoni* (black). The colors of the features in the splice junction evidence track correspond to the number of spliced RNA-Seq reads that support the splice junction (green: 50-99 reads; brown: 500-999 reads; red: ≥ 1000 reads). There is some evidence of alternative splicing within the 3' UTR as indicated by the green splice junctions JUNC00000556 and JUNC00000557 in comparison to the RefSeq model. However, these splice junctions are located outside the coding region, so they were not considered in our annotation. The Aggregated RNA-Seq track is the cumulative RNA-Seq read coverage from seven tissue-specific samples in *D. willistoni* males and seven tissue-specific samples in *D. willistoni* females. **
(B) Model of alignment of *D. melanogaster* and *D. willistoni Gar1* coding exons.** The two boxes depict the relative size and alignment of the coding exons of these two proteins. Exon boundaries are denoted by a color change and numbered sequentially. Notably, *D. melanogaster Gar1* has four coding exons while *D. willistoni Gar1* has three, and no coding exon boundaries appear to be conserved. **
(C) Comparison of genomic neighborhoods of *Gar1* in *D. melanogaster* Muller C element and putative *Gar1* in *D. willistoni* Muller F element.** Genes are depicted by arrows pointing in the direction of transcription. Nested gene pairs are depicted with a larger arrow containing a normally-sized gene arrow. Labels of *D. melanogaster* genes correspond to FlyBase annotations, whereas *D. willistoni* gene labels were assigned based on our annotation protocol, with the NCBI Gene ID listed below the gene symbol. Genes located on the Muller F element in *D. melanogaster* are orange. Genes located on the Muller C element in *D. melanogaster* are light blue. *Gar1* , in dark blue, is a C element gene in *D. melanogaster* that is nested within an intron of the *CG34396* gene. In contrast, *Gar1* in *D. willistoni* is flanked by F element genes and does not appear to be nested within the intron of a gene. **
(D) Graphic summary of the tBLASTn search of the *D. melanogaster* Gar1-PA protein (query) against the *D. willistoni* genome assembly (subject) without filtering for low-complexity regions.** The tBLASTn search identified 9,583 significant hits in 100 subject sequences of the *D. willistoni* genome assembly. tBLASTn alignments to the first 22 subject sequences are shown in the figure. The colors indicate alignment scores based on the BLOSUM62 matrix. The maroon box indicates the location of a match to the conserved domain H/ACA_rnp_Gar1/Naf1. There are very few hits that span the entire length of the *D. melanogaster* Gar1-PA protein sequence, with many only aligning to the ends of the protein, which is mostly outside the conserved domain. The first hit is the only red hit (alignment score > 200), which also covers almost the entire length of the *D. melanogaster* Gar1-PA protein. **
(E) Alignment of *D. melanogaster* Gar1-PA vs. *D. willistoni* Gar1-PA amino acid sequences.** Black lines in the dot plot indicate regions where the two sequences have identical amino acid residues. Regions with multiple short lines in the four corners of the dot plot indicate amino acids that are mapping to multiple locations in the *D. melanogaster* and *D. willistoni* proteins. Alternating background colors indicate coding exon boundaries. The maroon oval demarcates the conserved H/ACA_rnp_Gar1/Naf1 protein signature. Despite the major changes in gene structure, the length of Gar1-PA is similar between *D. melanogaster* and *D. willistoni* . **
(F) COBALT alignment of 12 *Drosophila* species** . Red boxes indicate aligned columns that are highly conserved across species and blue boxes indicate aligned columns that are less conserved according to the thresholds used by the “Conservation” coloring scheme in the NCBI Multiple Sequence Alignment Viewer. Grey boxes indicate aligned columns that include gaps in at least one species. The maroon box indicates the location of the known conserved domain H/ACA_rnp_Gar1/Naf1. The species are ordered by approximate phylogenetic distance, starting with the top three species in the melanogaster subgroup.

## Description

*Drosophila melanogaster* Gar1 ribonucleoprotein ( *Gar1)* gene (FlyBase accession FBgn0011824) encodes the Gar1 protein and is located on the right arm of chromosome 2 (chr2R; Muller C element). *Gar1* is assigned to the H/ACA Small Nucleolar Ribonucleoprotein Complex gene group in FlyBase (FBgg0001764). *Gar1* forms the H/ACA Small Nucleolar Ribonucleoprotein (snRNP) complex with *NHP2* (FBgn0029148), *Nop10* (FBgn0033548), and *Nop60B* (FBgn0259937) (Breznak et al., 2023, Sanchez et al., 2016). This complex is vital for ribosome biogenesis, forming a complex that assists in the pseudouridylation of specific sites in ribosomal RNA (Breznak et al., 2023, Sanchez et al., 2016). *Gar1* contains the H/ACA ribonucleoprotein complex, subunit Gar1/Naf1 (H/ACA_rnp_Gar1/Naf1) protein signature (of the protein family IPR007504), which identifies protein domains that bind to *Cbf5* (Leulliot et al., 2007). The conserved protein signature is flanked on both sides by glycine-rich regions. According to the “Orthologs” section of the FlyBase *Gar1* gene report (FBgn0011824), *D. melanogaster Gar1* is orthologous to human *GAR1* (NCBI Gene ID 54433).

**Large number of changes in gene structure compared to the *D. melanogaster* ortholog**

A preliminary FlyBase BLAST (database release 6.53) search of the *D. willistoni* protein sequence XP_002072577.1 (derived from the gene with the NCBI Gene ID 6651170) to the *D. melanogaster* annotated proteins database yields only one hit to Gar1-PA with a score of 215.698 and an E-value of 3e-56, prompting an initial ortholog assignment of *Gar1* . The *D. willistoni Gar1* gene model was annotated according to the protocol detailed in Rele et al., 2023. The *Gar1* gene in *D. melanogaster* contains one isoform (Gar1-PA) with three coding exons. In contrast, the proposed gene model for *D. willistoni* has four coding exons ( Figure 1A ). The gene structure is supported by multiple lines of experimental and computational evidence. For example, all donor and acceptor sites are supported by both significant drops in the aggregated RNA-Seq expression data coverage and corresponding splice junctions. The chosen start codon is the only option that maintains an open reading frame in coding exon 1 without truncating conserved amino acids from the N-terminus. Compared to *D. melanogaster* , *Gar1* has multiple complex changes in gene structure ( Figure 1B ). The first coding exon of the *D. willistoni* Gar1-PA ortholog aligns to the first coding exon in *D. melanogaster* as well as the first 111 amino acids of the second coding exon in *D. melanogaster* . The second coding exon of *D. willistoni* aligns to amino acids 112-125 of the second coding exon in *D. melanogaster.* The third coding exon of *D. willistoni* aligns to the last amino acid of the second coding exon as well as the first 12 amino acids of the third coding exon in *D. melanogaster.* The fourth coding exon of *D. willistoni* aligns to amino acids 13-47 of the third coding exon in *D. melanogaster* .

***Gar1* is located on a different chromosome and in a different genomic neighborhood than the *D. melanogaster* ortholog**

In *D. melanogaster* , *Gar1* is located on chr2R, which corresponds to the Muller C element, whereas our gene of interest is located on chromosome 3 (Muller E+F elements) in *D. willistoni* . Moreover, local synteny analysis reveals that the gene is located in different genomic neighborhoods in the two species ( Figure 1C ). In *D. willistoni* , *Gar1* is flanked by F element genes rather than the C element genes surrounding the *D. melanogaster* ortholog. Additionally, in *D. melanogaster* , *Gar1* is nested within an intron of CG *34396* (Gene ID: 37398). There is insufficient evidence based on our published annotation protocol to suggest that the putative *Gar1* gene in *D. willistoni* is nested within a gene, *CG34396* or otherwise. In *D. willistoni* the ortholog of *CG34396* is found at a single locus within the *D. willistoni* genome on scaffold NW_025814046. This scaffold has tentatively been assigned to the Muller C element.

***D. willistoni* feature with NCBI Gene ID 6651170 putative orthology assigned to *Gar1* .**

The large changes in gene structure of the proposed *Gar1* gene and its new chromosomal environment prompted a deeper investigation into the orthology assignment. A tBLASTn search (BLAST+ 2.16.0) with the *D. melanogaster* Gar1-PA protein sequence (FBpp0071562) against the entire *D. willistoni* genome assembly (GCF_018902025.1) was performed using the search parameters as described in Rele et al., 2023, without masking for low complexity regions. The top hit is to scaffold NC_061086.1 (chromosome 3) with a max score of 301, total score of 36,382, and an E-value of 3e-93, in comparison to the second-best hit to scaffold NW_025814363.1 with a lower max score of 108, lower total score of 19,400, and a higher E-value of 6e-27. The top individual hit within chromosome 3 maps to the expected *D. willistoni* feature (NCBI Gene ID 6651170, XM_002072541) with E-value 3e-93 and percent identity 65%. Additionally, the graphic summary ( Figure 1D ) demonstrates that many of the other top hits to the query sequence only align to the ends of the protein (low complexity, glycine-rich regions) rather than the central region with the conserved domain IPR007504. Repeating the tBLASTn search with the low complexity filter yields just one hit to the feature of interest in chromosome 3 and has coordinates that encompass the conserved domain. This hit has a score of 185, an E-value of 9e-53, and percent identity of 92%. Because both tBLASTn searches (with and without the low complexity filter) placed the best match to the *D. melanogaster Gar1* gene in the same genomic region, the feature is likely to be the ortholog, rather than a novel paralog, of *Gar1* . The low E-values and high percent identities for both whole genome tBLASTn searches further support the hypothesis that the feature is the putative ortholog despite the major changes in gene structure.

***Gar1* includes a conserved domain and glycine-rich ends**

A dot plot of the alignment between the Gar1-PA proteins in *D. melanogaster* and *D. willistoni* ( Figure 1E ) depicts a region of high sequence similarity as demarcated by the maroon oval, which contains the match to the H/ACA_rnp_Gar1/Naf1 protein signature (IPR007504). Outside of this region there is a high density of short alignment blocks, which can be attributed to multiple matches to the glycine-rich regions at the N-terminus and C-terminus of the Gar1-PA protein. The COBALT multiple sequence alignment ( Figure 1F ) demonstrated high conservation of the H/ACA_rnp_Gar1/Naf1 conserved domain across twelve *Drosophila* species. The low-complexity regions on the N and C-terminus show comparatively lower conservation, which demonstrates the potential for variability in these regions and is consistent with the importance of the conserved domain to *Gar1* function.

## Methods

Annotation of protein-coding genes was performed according to the protocol in Rele et al., 2023, based on FlyBase *D. melanogaster* gene models in version 2024_06 ( *D. melanogaster* release 6.53), and using FlyBase BLAST version 2.2.18. The protocol included accessing a version of the UCSC Genome Browser created by Genomics Education Partnership (GEP) (Genomics Education Partnership, 2025). Additionally, the Gene Record Finder (Genomics Education Partnership, 2025) was used to find isoform and coding exon information in *D. melanogaster* and the Gene Model Checker (Genomics Education Partnership, 2025) was used to produce an alignment of *D. willistoni* and *D. melanogaster* Gar1-PA proteins ( Figure 1E ). The *D. willistoni* genome (UCI_dwil_1.1: GCF_018902025.1; GCA_018902025.2) was viewed in the Genome Browser with experimental and computational evidence tracks, including RNA-Seq (PRJNA388952, SRP108530), tBLASTn (BLAST+ 2.16.0), *D. willistoni* RefSeq genome assembly (PRJNA811224), and Genscan computational gene predictor. NCBI BLAST+ v2.16.0 was used to compare *D. melanogaster* and *D. willistoni* sequences using no compositional score adjustment. Otherwise, default parameters were used. Figure 1F was generated using the Constraint-based Alignment Tool (COBALT) (Papadopoulos & Agarwala, 2007). The website version of COBALT was used to perform the analysis (last accessed on February 21st, 2025). Unless otherwise stated, algorithm versions and assembly accession numbers are consistent with Rele et al., 2023. The Aggregated RNA-Seq track shows the cumulative RNA-Seq read coverage from seven tissue-specific samples in *D. willistoni* males and females. These tissue types include male head, male thorax, male abdomen, male digestive system, male gonad, male reproductive tract, and male whole body, as well as female head, female thorax, female abdomen, female digestive system, female gonad, female reproductive tract, and female whole body (NCBI SRA accession number SRP108530) (Yang et al., 2018). The RNA-Seq data from these tissues were combined into one track using bigWigMerge, one of the command line utilities provided by the UCSC Genome Browser Project Team (Perez et al., 2025).

## Data Availability

Description: Gar1 Annotation Files. Resource Type: Model. DOI: https://doi.org/10.22002/1jkr9-50n69 Description: Gar1 Protein Sequence Alignment. Resource Type: Image. DOI: https://doi.org/10.22002/753g7-4re52
